# Effects of Concentrates with Different Energy and Protein Levels on Growth Performance, Blood Metabolism, and Rumen Microbiota of Housed Pregnant Yaks During the Warm Season

**DOI:** 10.3390/microorganisms14071506

**Published:** 2026-07-10

**Authors:** Zhenyu Zhu, Qunying Zhang, Fuju Chen, Yingkui Yang, Binqiang Bai, Yan Bai, Deyu Yang, Chengxiang Ding, Lizhuang Hao, Jianbo Zhang

**Affiliations:** Qinghai Academy of Animal Sciences and Veterinary Medicine, Key Laboratory of Plateau Grazing Animal Nutrition and Feed Science of Qinghai Province, Qinghai University, Qinghai 810016, China; mrzhu2024@outlook.com (Z.Z.); zqy1010307564@126.com (Q.Z.);

**Keywords:** pregnant yak, housed feeding, concentrate nutrition level, growth performance, blood metabolism, rumen microbiota

## Abstract

The nutritional management of pregnant yaks is critical yet remains understudied, particularly under housed conditions. This study investigated the effects of concentrate supplementation at varying nutritional levels on growth performance, blood metabolism, and rumen microbiota in housed pregnant yaks during the warm season. Eighteen mid-gestation yaks were allocated to low- (LP), medium- (MP), and high-nutrition (HP) groups and fed for 110 days. Growth performance, serum metabolites, hormones, and rumen microbiota composition were assessed. No significant differences were observed in final body weight, average daily gain, or dry matter intake among the groups (*p* > 0.05). However, the serum malondialdehyde concentration was significantly higher in the MP group (3.03 nmol/mL) than in the LP group (1.30 nmol/mL) (*p* < 0.05). Concentrations of glucose and lipid metabolites tended to increase with higher nutritional levels, whereas circulating levels of growth-related hormones (GH, INS, IGF-1, and IGF-2) showed a declining trend (*p* > 0.05). Rumen alpha diversity was numerically highest in the MP group. Specific taxa (*norank_o_Clostridia_UCG-014*) were enriched in the MP and LP groups (*p* < 0.05). The microbial composition in the MP group was positively correlated with blood TG, TC, and HDL-C (*p* < 0.05). In conclusion, within the constraints of the present experimental design, medium- or low-nutrition concentrate supplementation maintained growth performance and modulated the rumen microbiota associated with lipid metabolism. However, the elevated MDA in the MP group warrants caution, and the observed trends should be interpreted as preliminary and require validation in future studies with larger sample sizes.

## 1. Introduction

The yak (*Bos grunniens*) is a unique ruminant endemic to the Qinghai–Tibetan Plateau. Through long-term natural selection and domestication, yaks have developed remarkable adaptations to high-altitude and cold environments. They are not only a vital component of the plateau ecosystem but also a crucial livestock species for sustaining the livelihoods of local herders [1,2]. Under traditional grazing systems, however, yaks often face a cyclical pattern of “summer thriving, autumn fattening, winter thinning, and spring weakness” due to seasonal fluctuations in forage availability on the alpine grasslands [3,4]. This seasonal nutritional stress not only restricts individual growth but also, more critically, leads to widespread undernutrition in pregnant yaks, which adversely affects calf health and herders’ economic returns. Therefore, establishing science-based nutritional strategies and feeding standards for different production systems is essential for the sustainable development of yak husbandry.

Research on yak nutrition has progressed in recent years. Studies indicate that appropriate concentrate supplementation during the cold and warm seasons can promote the proliferation of rumen bacteria involved in volatile fatty acid production, enhances fermentation function, and improves the growth performance of grazing yaks [5,6]. The energy and protein levels of the supplement are key factors [6,7], and feeding management practices also play a significant role [8]. Supplementation during gestation is particularly important, since it can improve calving rates and enhance nutrient supply to the fetus in late pregnancy and to the neonate in early lactation. These improvements contribute to increased calf birth weight and elevated levels of growth-related hormones and immunoglobulins, thereby promoting calf development [9,10,11]. Nevertheless, systematic research on the nutritional requirements of pregnant yaks remains limited compared with that on other livestock species. Most existing supplementation studies have focused on grazing yaks or fattening periods [5,6,12], with few reports on how the nutritional level of concentrates regulates the rumen microbiota of housed pregnant yaks and subsequently influences their performance. Furthermore, the interaction between dietary nutritional levels and the rumen microbiome, and how this interaction subsequently affects host metabolism and performance, remains poorly understood in pregnant yaks under barn feeding conditions.

Mid-gestation represents a critical physiological window for fetal organogenesis and placental development. During this stage, maternal metabolism undergoes a transition, shifting its priority towards supporting fetal growth. Specifically, the rapid development of fetal organs and the formation of the placental vascular system impose substantially increased demands for energy, protein, and specific micronutrients. Maternal metabolic adaptation during this period involves enhanced gluconeogenesis, altered lipid metabolism, and increased amino acid mobilization to support fetal requirements [13,14]. Furthermore, the energy and protein demands of the pregnant yak increase significantly during mid-gestation; deficiencies can readily lead to fetal growth retardation and maternal metabolic disturbances [10].

To address these research gaps, the present study was designed with three concentrate nutritional levels (low, medium, and high) based on graded digestible energy (DE: 11.92, 13.14, and 14.23 MJ/kg) and crude protein (CP: 18.04, 19.89, and 21.87%) contents, with reference to the Chinese Feeding Standard for Beef Cattle (NY/T 815-2004) [15]. The gradient design was intended to identify the optimal supplementation level for housed pregnant yaks during the warm season by evaluating dietary nutritional inputs. Therefore, this study investigated housed pregnant yaks supplemented with concentrates at low-, medium-, and high-nutritional levels. By integrating analyses of growth performance, blood metabolic parameters, and rumen microbial composition, we aimed to: (1) elucidate the effects of different concentrate nutritional levels on production performance and metabolic status in housed pregnant yaks; and (2) explore the underlying microbiological mechanisms linking dietary nutrition to host physiological responses. The core innovations of this study are twofold: first, the integrated analysis of production performance, blood metabolism, and rumen microbial community structure to comprehensively evaluate nutritional effects; and second, the systematic screening of the optimal concentrate nutritional range for housed pregnant yaks during the warm season, providing a scientific basis for precision feeding strategies on the Qinghai–Tibet Plateau. The findings are expected to provide a scientific basis for the precise nutritional management of pregnant yaks.

## 2. Materials and Methods

### 2.1. Experimental Design

The animal trial protocol was approved by the Laboratory Animal Welfare and Ethics Review Committee of the Qinghai Academy of Animal Science and Veterinary Medicine (Permit Number: 2025-QHMKY-020). The experiment was conducted from April to August 2025 at Qinghai Zhonglei Biotechnology Co., Ltd., Maqin County, Golog Tibetan Autonomous Prefecture, Qinghai Province, China. During the trial (April to August 2025), the average ambient temperature was approximately 8.0 °C. Monthly mean temperatures increased progressively from 2.5 °C in April to 13.0 °C in July, and then reached 14.5 °C in August. The overall average relative humidity was 65%.

Eighteen healthy, mid-gestation (approximately 100 days) pregnant yaks of similar age and body weight (227.78 ± 11.05 kg) were selected based on artificial insemination records and B-ultrasound examination. They were randomly assigned to three treatment groups: low-nutrition (LP), medium-nutrition (MP), and high-nutrition (HP). Each group consisted of 6 yaks, with one yak housed in a separate pen. Individual feed intake was recorded for each yak, and each animal served as the experimental unit for all measured variables. The trial included a 7-day adaptation period followed by a 110-day formal feeding period. During the 7-day adaptation period, the yaks were gradually transitioned from the basal diet (oat hay alone) to the experimental diets by incrementally increasing the concentrate proportion over the first 4 days, followed by 3 days of full adaptation to the target concentrate-to-roughage ratio (4:6). The 7-day adaptation period was chosen based on previous yak studies, which demonstrated that this duration is sufficient for yaks to acclimate to dietary transitions under housed conditions, as the basal diet was familiar to the animals and the concentrate-to-roughage ratio was consistent across all groups. However, we acknowledge that a longer adaptation period may be necessary for complete rumen microbial stabilization. Throughout the trial, the yaks were monitored daily for feed intake to ensure health and welfare. The yaks were housed in individual pens and fed twice daily (at 07:00 and 16:00). At each feeding, the experimental diets were weighed and provided according to the designated concentrate-to-roughage ratio (4:6, dry matter basis). All yaks had ad libitum access to feed and water throughout the trial. All yaks were fasted for 12 h before their initial and final body weights were measured. Pen hygiene was maintained through regular cleaning, and ventilation was provided.

### 2.2. Experimental Diets

All yaks were fed a basal diet of oat hay. The nutritional composition of the oat hay is provided in Supplementary Table S1. Concentrate supplements were offered at a concentrate-to-roughage ratio of 4:6 (dry matter basis). The concentrates were formulated according to the nutrient recommendations for beef cattle specified in the Chinese Feeding Standard for Beef Cattle (NY/T 815-2004) [15]. They consisted of corn, corn germ meal, wheat middlings, corn DDGS, soybean meal, cottonseed meal, rapeseed meal, dicalcium phosphate, and an additive premix. The detailed composition and analyzed nutritional levels of the three concentrates, with DE values of 11.92, 13.14, and 14.23 MJ/kg and CP contents of 18.04%, 19.89%, and 21.87%, respectively, are presented in Table 1. Actual daily nutrient intakes (DE and CP) based on the measured DMI are provided in Supplementary Table S1.

### 2.3. Determination of Growth Performance

Initial body weight (IBW) was measured on the morning of the first day of the formal trial, and final body weight (FBW) was measured on the morning of day 120, both prior to the morning feeding. Average daily gain (ADG) was calculated as: ADG (kg/d) = (FBW − IBW)/trial days. Total feed intake for each animal was recorded throughout the trial to calculate average daily dry matter intake (DMI) and the feed-to-gain ratio (F/G): DMI = total dry matter intake/trial days; F/G = DMI/ADG.

### 2.4. Sample Collection

At the end of the trial, before the morning feeding, blood samples (approximately 10 mL) were collected from the jugular vein of each yak into vacuum tubes without anticoagulant. After clotting at room temperature for 30 min, the samples were centrifuged at 1000× *g* for 15 min at 4 °C. The resulting serum was aliquoted into 1.5 mL tubes and stored at −20 °C for subsequent analysis. Rumen fluid samples were collected simultaneously via a stomach tube before the morning feeding. After discarding the first 50 mL of fluid to avoid saliva contamination, approximately 30–50 mL of rumen fluid was collected into a sterile tube, immediately filtered through four layers of sterile gauze, and the filtrate was aliquoted into 2 mL cryotubes. The samples were immediately frozen in liquid nitrogen and subsequently stored at −80 °C for DNA extraction and microbiota analysis.

### 2.5. Blood Biochemical Indicator Testing

Serum concentrations of glucose (GLU), triglycerides (TG), total cholesterol (TC), high-density lipoprotein cholesterol (HDL-C), and low-density lipoprotein cholesterol (LDL-C) were measured using an automatic biochemical analyzer (Mindray BS-240VET, Shenzhen, China) according to standard colorimetric methods. Concentrations of non-esterified fatty acids (NEFA), total antioxidant capacity (T-AOC), malondialdehyde (MDA), and glutathione peroxidase (GSH-Px) were determined using commercial assay kits (Nanjing Jiancheng Bioengineering Institute, Nanjing, China) following the manufacturer’s instructions by colorimetric and enzymatic methods. Serum NEFA concentrations were measured using an ELISA kit (Nanjing Jiancheng Bioengineering Institute, Nanjing, China) according to the manufacturer’s protocol. Serum concentrations of growth hormone (GH), insulin (INS), insulin-like growth factor 1 (IGF-1), and insulin-like growth factor 2 (IGF-2) were quantified using commercially available enzyme-linked immunosorbent assay (ELISA) kits (Zhuocai Biotechnology, Shanghai, China).

### 2.6. Detection of Rumen Microbial Community Diversity and Bioinformatics Analysis

Genomic DNA was extracted using the CTAB method. The quality and concentration of the extracted DNA were assessed via 1.0% agarose gel electrophoresis and a NanoDrop^®^ ND-2000 spectrophotometer (Thermo Fisher Scientific, Waltham, MA, USA). DNA samples were then stored at −80 °C until further analysis. To analyze the bacterial community composition, the V5-V7 region of the 16S rRNA gene was amplified using the primers 799F (5′-AACMGGATTAGATACCCKG-3′) and 1193R (5′-ACGTCATCCCCACCTTCC-3′), with a unique 6 bp sample barcode added to differentiate the samples. The PCR reaction system (25 µL total volume) consisted of 12.5 µL of 2× Phusion Master Mix, 1.0 µL of each primer (10 µM), 2.0 µL of template DNA (20 ng/µL), and 8.5 µL of nuclease-free water. The PCR conditions were as follows: initial denaturation at 98 °C for 1 min, followed by 30 cycles of denaturation at 98 °C for 10 s, annealing at 50 °C for 30 s, and extension at 72 °C for 30 s, with a final extension at 72 °C for 5 min. PCR products were purified and quantified using a Qiagen Gel Extraction Kit (Qiagen, Hilden, Germany). The products were separated on a 2% agarose gel, purified, and quantified using a Synergy HTX microplate reader (Biotek, Winooski, VT, USA). The purified amplicons were then pooled in equimolar amounts and sequenced using paired-end sequencing on an Illumina NextSeq 2000 PE300 platform (Illumina, San Diego, CA, USA).

Raw paired-end sequences were imported into QIIME2 (version 2022.2) for quality control, assembly, and generation of amplicon sequence variants (ASVs) using the default workflow. Raw reads were quality-filtered by removing reads with average quality scores below Q20, discarding reads shorter than 200 bp, and removing chimeric sequences using the VSEARCH algorithm. ASVs were clustered with 100% sequence identity, and ASVs with an abundance of less than 0.001% of total reads were removed. Bacteria were classified and annotated using the Silva database (version 138.1) (https://www.arb-silva.de (accessed on 15 November 2025)). QIIME2 was used to estimate species richness (Chao1 index) and the Shannon diversity index. Non-parametric Kruskal–Wallis tests were used to analyze the alpha diversity of rumen microbial communities [16]. Non-metric multidimensional scaling (NMDS) based on Bray–Curtis distances was used to analyze beta diversity, and ANOSIM tests were used to assess differences among groups [17]. Circos software (version 0.69-9) was utilized for the visual analysis of rumen microbial community composition among groups [18]. Linear discriminant analysis coupled with effect size (LEfSe) was used to identify differentially abundant taxa among groups, with a threshold of an LDA score > 2 and *p* < 0.05. Mantel tests were conducted to analyze the correlations between rumen microbial communities and blood biochemical indicators.

### 2.7. Statistical Analysis

All data were first tested for normality using the Shapiro–Wilk test and for homogeneity of variance using Levene’s test in R software (version 4.3.1). For normally distributed phenotypic data (growth performance and blood biochemical indices), one-way ANOVA was performed, followed by the Least Significant Difference (LSD) test for multiple comparisons. For rumen microbial data, which did not conform to a normal distribution, non-parametric Kruskal–Wallis tests were used for alpha diversity comparisons and differential taxa identification (*n* = 6). Results are presented as the mean ± standard error of the mean (SEM). A *p* < 0.05 was considered to indicate a significant difference, *p* < 0.01 was considered a highly significant difference, and *p* > 0.05 was considered no significant difference.

## 3. Results

### 3.1. The Effects of Different Nutritional Levels of Supplementary Feed on the Production Performance and Blood Metabolites of Pregnant Yaks

Final body weight and average daily gain did not differ significantly among groups despite the increasing nutritional level of the concentrate feed. By the end of the experiment, body weight had increased by 27.50%, 26.16%, and 27.34% in the LP, MP, and HP groups, respectively (Table 2, *p* > 0.05). Although the numerical values of ADG were similar across groups (0.57, 0.54, and 0.56 kg/d for the LP, MP, and HP groups, respectively), the lack of statistical significance indicates that the dietary treatments did not exert a detectable effect on growth performance under the current experimental conditions. Average daily dry matter intake gradually decreased as the concentrate nutritional level increased. However, neither the feed-to-gain ratio nor feed conversion efficiency differed significantly among groups (*p* > 0.05). Calculated daily DE and CP intakes based on the actual DMI are presented in Supplementary Table S1.

With the increase in the nutritional level of the concentrate feed, the concentrations of serum indicators related to glucose and lipid metabolism, such as GLU, TC, HDL, and LDL, gradually increased across the LP, MP, and HP groups, but these differences were not significant (*p* > 0.05). However, serum MDA concentration was significantly higher in the MP group than in the LP group (*p* < 0.05). With the increase in the nutritional level of the concentrate feed, the concentrations of serum growth-related hormones, such as GH, INS, IGF-1, and IGF-2, in the LP, MP, and HP groups showed a downward trend (GH: 2.97, 2.40, and 1.98 ng/mL; INS: 4.64, 4.39, and 3.52 mIU/L; IGF-1: 48.90, 44.19, and 32.67 ng/mL; IGF-2: 7.06, 6.63, and 5.06 ng/mL for LP, MP, and HP, respectively), but there were no significant differences among the groups (*p* > 0.05).

### 3.2. The Impact of Different Nutritional Levels of Supplementary Feed on the Diversity of Rumen Microbial Communities in Pregnant Yaks

**Figure 1:**
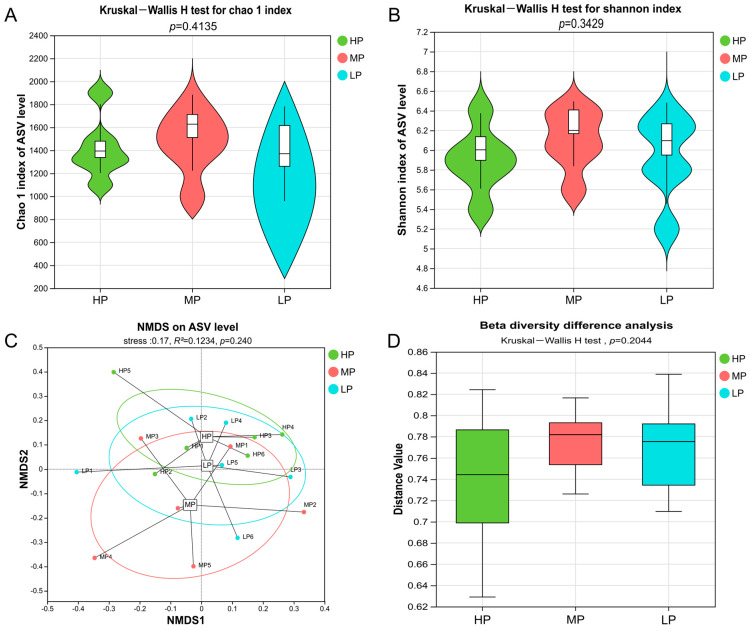
Alpha and beta diversity of the rumen microbiota in pregnant yaks fed concentrates at different nutritional levels. (**A**) Chao1 index. (**B**) Shannon index. (**C**) Non-metric multidimensional scaling (NMDS) plot based on Bray–Curtis distances. (**D**) Beta diversity difference analysis. LP, low-nutrition group; MP, medium-nutrition group; HP, high-nutrition group (*n* = 6).

With the increase in the nutritional level of the concentrate feed, there were no significant differences in the Chao1 species richness index or the Shannon diversity index of the rumen microbial community in pregnant yaks (Figure 1A,B, Kruskal–Wallis test, *p* > 0.05). Furthermore, neither the alpha diversity nor the beta diversity of the rumen microbial community differed significantly among the three groups (*p* > 0.05). Non-metric multidimensional scaling (NMDS) ordination plots based on Bray–Curtis distances were used to further visualize the impact of different nutritional levels of concentrate feed on rumen microbial community structure. The results showed that the effects were not significant (Figure 1C, ANOSIM test, *p* > 0.05), although the MP group was positioned farther from the other two groups. Similarly, analysis based on the Kruskal–Wallis test indicated that the samples from the MP group were farther away from the samples of the other two groups, and there were no significant differences in the structure of the rumen microbial community among the groups (Figure 1D, *p* > 0.05).

### 3.3. The Impact of Different Nutritional Levels of Supplementary Feed on the Composition of Rumen Microbial Communities in Pregnant Yaks

**Figure 2:**
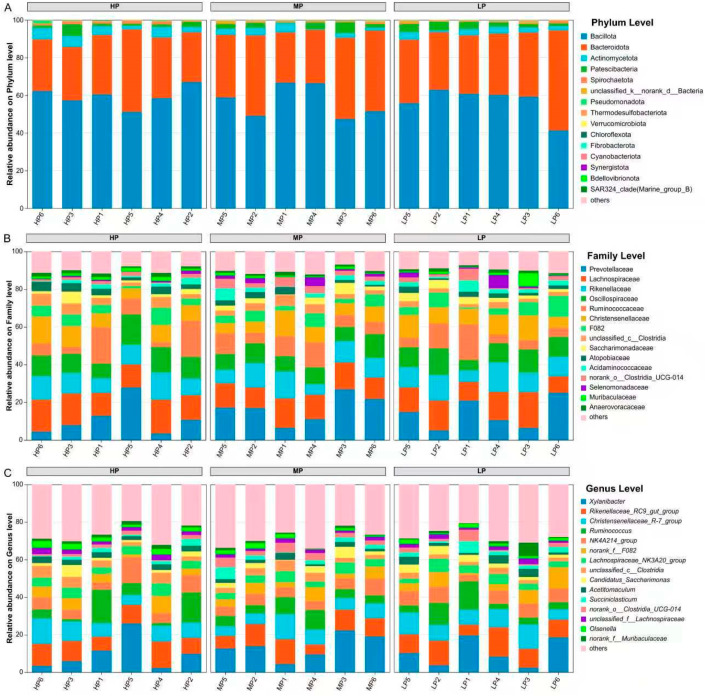
Composition of the rumen microbiota in pregnant yaks at different taxonomic levels. (**A**) Phylum level. (**B**) Family level. (**C**) Genus level. Only the top 15 most abundant taxa are shown at each level; others are grouped as “Other”, which represents low-abundance unclassified taxa with individual relative abundances below 1% that were not individually labeled. LP, low-nutrition group; MP, medium-nutrition group; HP, high-nutrition group (*n* = 6).

Taxonomic annotation at different levels revealed that the dominant phyla across groups were Firmicutes (40.0–70.0%), Bacteroidota (18.5–35.3%), and Actinomycetota (3.1–4.2%) (Figure 2A). As shown in Figure 2B, the dominant families in the rumen microbial communities of pregnant yaks across the different groups were *Prevotellaceae*, *Lachnospiraceae*, *Rikenellaceae*, *Oscillospiraceae*, *Ruminococcaceae*, and *Christensenellaceae*. At the genus level, *Xylanibacter*, *Rikenellaceae_RC9_gut_group*, *Christensenellaceae_R-7_group*, and *Ruminococcus* were commonly found in pregnant yaks across the different groups (Figure 2C).

### 3.4. Analysis of Differences in Rumen Microbial Communities of Pregnant Yaks with Different Nutritional Levels of Supplemental Feed

**Figure 3:**
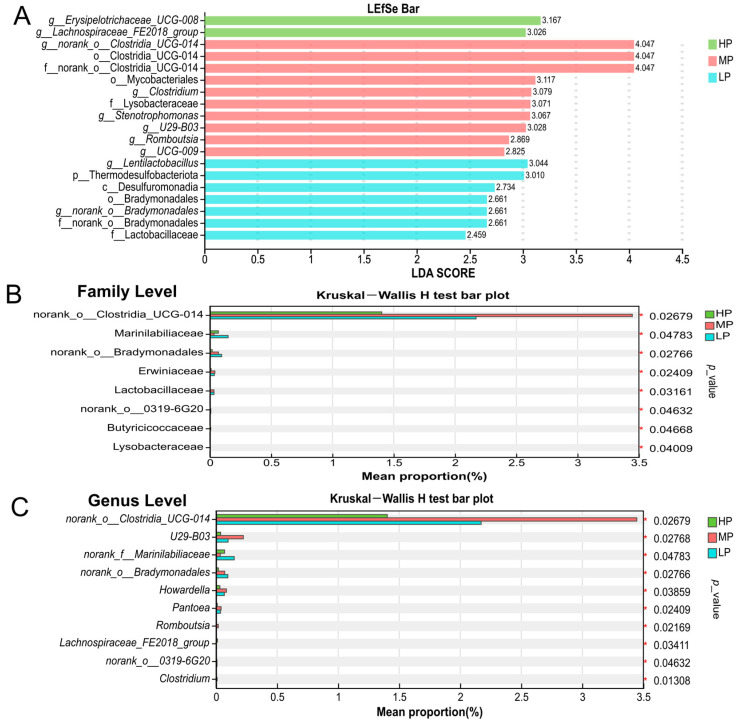
Differential abundance analysis of rumen microbiota. (**A**) Cladogram generated from LEfSe analysis showing the phylogenetic distribution of microbial taxa enriched in different groups. (**B**,**C**) Relative abundance of significantly different microbial taxa at the family (**B**) and genus (**C**) levels as determined by Kruskal–Wallis test * *p* < 0.05 (*n* = 6).

To further determine the impact of different nutritional levels on the rumen microbial community, linear discriminant analysis effect size (LEfSe) was used to identify differentially abundant taxa among the groups (Figure 3A). The LEfSe results showed that the MP group was enriched with *norank_o_Clostridia_UCG-014*, *Lysobacteraceae*, *Clostridium*, *Stenotrophomonas*, and *Romboutsia* (*p* < 0.05), whereas the LP group was enriched with *Lentilactobacillus* (*p* < 0.05). Furthermore, the Kruskal–Wallis rank sum test confirmed that, compared with the HP group, the MP and LP groups were significantly enriched with *norank_o_Clostridia_UCG-014*, *norank_o_Bradymonadales*, *Erwiniaceae* and *Lactobacillaceae* (Figure 3B, *p* < 0.05), as well as *U29-B03*, *Howardella*, and *Pantoea* (Figure 3C, *p* < 0.05).

### 3.5. Analysis of Factors Influencing the Structural Changes of the Rumen Microbiota in Pregnant Yaks

**Figure 4:**
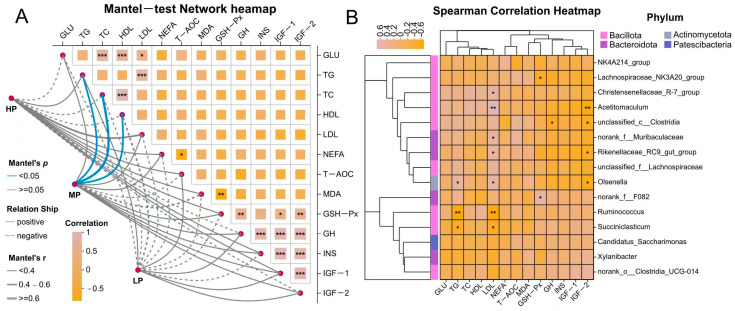
Correlations between rumen microbiota and host serum parameters. (**A**) Mantel test showing the correlation between the overall genus-level microbial community composition and serum parameters for each group. Line width represents the Mantel’s r statistic, and color indicates significance. (**B**) Heatmap of Spearman correlation coefficients between the relative abundance of the top 20 genera and serum parameters. * *p* < 0.05, ** *p* < 0.01, *** *p* < 0.001 (*n* = 6).

Spearman correlation and Mantel tests were used to assess the relationship between rumen microbial community composition (at the genus level) and host blood metabolites. Host blood GLU was significantly positively correlated with TC, HDL, and LDL (*p* < 0.05), GH was significantly positively correlated with INS, IGF1, and IGF2 (*p* < 0.05), and INS was significantly positively correlated with IGF1/ IGF2 (*p* < 0.05). Additionally, MDA was significantly negatively correlated with GSH-Px (*p* < 0.05). At the genus level (Figure 4B), TG and LDL concentrations were significantly negatively correlated with the relative abundance of *Ruminococcus* and *Succiniclasticum* (*p* < 0.05) but positively correlated with *Olsenella* (*p* < 0.05). LDL was significantly positively correlated with *Christensenellaceae_R-7_group*, *Acetitomaculum*, *norank_f_Muribaculaceae*, and *Rikenellaceae_RC9_gut_group* (*p* < 0.05), whereas IGF-2 was significantly negatively correlated with *Acetitomaculum*, *unclassified_c_Clostridia*, *Rikenellaceae_RC9_gut_group*, and *Olsenella* (*p* < 0.05).

## 4. Discussion

As a ruminant unique to the Qinghai–Tibet Plateau, the yak faces a persistent contradiction between its physiological adaptability and seasonal nutritional deficiencies—a core issue in plateau animal husbandry research [2]. Research on the nutrition of pregnant yaks still lags far behind that of other domestic animals. Affected by the harsh natural environment of the plateau, particularly in winter and spring when temperatures are low, snowfall is heavy, and grassland forage is depleted, yaks graze without supplementary feeding. This situation is detrimental to the growth and development of pregnant yaks and their fetuses, adversely affects parturition, causes frequent dystocia, and delays the recovery of postpartum body condition, the return of estrous cycles, and normal lactation. Therefore, in order to address the nutritional deficiencies of pregnant yaks and thereby improve their production performance, supplementary feeding for pregnant yaks is especially important in the cold season and is a very effective strategy.

### 4.1. Effects of Concentrate Supplementation on the Production Performance of Pregnant Yaks

Previous research has predominantly focused on supplementation strategies for grazing yaks during the cold season. In contrast, systematic investigations into how varying nutritional levels in concentrate supplements modulate the rumen microbiota and, subsequently, the production performance of housed pregnant yaks remain scarce. This study adopted an integrative approach, combining analyses of production performance, blood metabolic parameters, and rumen metagenomics to elucidate the mechanistic responses of housed pregnant yaks to increasing dietary nutritional levels. At the trial’s conclusion, final body weights had increased by 27.50%, 26.16%, and 27.34% in the LP, MP, and HP groups, respectively. However, no significant differences were observed in final body weight or average daily gain among the groups. This phenomenon likely reflects a fundamental physiological adaptation in pregnant ruminants, whereby nutrients are prioritized for fetal development over maternal weight gain to meet the escalating demands of gestation. Reynolds et al. [13] also noted that late-gestation cows often enter a state of negative energy balance, with ingested nutrients preferentially allocated to support fetal protein and fat deposition. Furthermore, yaks, as ruminants exquisitely adapted to high-altitude environments, may possess more conservative energy metabolism and nutrient partitioning mechanisms. Under the stable conditions of the warm season in this study, the energy provided even by the low-nutrition concentrate may have been sufficient to meet their basal maintenance requirements. This view is supported by studies by Yang et al. [7] and Dai et al. [5], which similarly indicated that once dietary energy meets an adequate threshold, further increments do not translate into significant improvements in average daily gain. The numerical decrease in DMI (from 5.58 to 5.15 kg/d) with increasing dietary nutritional levels further supports the concept of energy intake regulation, whereby yaks reduced their feed consumption to maintain a relatively constant nutrition intake across groups. Consequently, from a practical feeding standpoint, excessively high-nutritional levels in concentrate supplements appear unnecessary for pregnant yaks during the warm season; medium- to low-nutritional levels are sufficient to meet their gestational needs. These findings provide a valuable scientific basis for implementing precise nutritional strategies for gestating yaks in high-altitude production systems. The absence of significant differences in growth performance among the three nutritional treatments has important practical implications. From a production perspective, low- or medium-nutrition concentrates appear sufficient to support the growth and gestational needs of housed pregnant yaks during the warm season, whereas high-nutrition concentrates provide no additional benefit in terms of weight gain. This suggests that feeding high-nutrition concentrates to housed pregnant yaks during the warm season may be economically unnecessary, as it increases feed costs without improving performance. These findings support the development of cost-effective precision feeding strategies for yak production systems on the Qinghai–Tibet Plateau.

### 4.2. Effects of Concentrate Supplementation on Blood Metabolism in Pregnant Yaks

Animal growth is governed by a complex interplay of genetic, nutritional, and environmental factors, with blood metabolites playing a pivotal regulatory role [19]. Nutrients from feed are digested, absorbed into the bloodstream, and subsequently metabolized and deposited in target tissues. Therefore, serum biochemical parameters serve as crucial indicators of an animal’s physiological status and overall health [20,21]. Blood glucose concentration serves as an indicator of systemic energy metabolism, whereas triglycerides, total cholesterol, low-density lipoprotein cholesterol, high-density lipoprotein cholesterol, and non-esterified fatty acids (NEFA) are key markers of energy balance and lipid metabolism [22]. In the present study, serum parameters related to glucose and lipid metabolism exhibited an increasing trend across the LP, MP, and HP groups with increasing concentrate nutritional levels, although these trends did not reach statistical significance. This suggests that appropriate concentrate supplementation can adequately support the physiological and production demands of housed yaks during gestation in the warm season. For yaks, elevating dietary energy levels is known to enhance growth performance and modulate lipid metabolism [23]. Furthermore, we found that the serum MDA concentration, an indicator of lipid peroxidation, was significantly lower in the LP group compared to the MP group (*p* < 0.05). However, the significantly elevated MDA in the MP group (3.03 nmol/mL) compared with that in the LP group (1.30 nmol/mL) points toward greater oxidative stress at the medium nutritional level. This non-linear pattern suggests that the MP group may have experienced a transient metabolic challenge that was not fully compensated for by the antioxidant system, as T-AOC and GSH-Px did not differ significantly among the groups (*p* > 0.05). Serum antioxidant profiles reflect the body’s capacity to counteract oxidative stress. T-AOC provides a composite measure of overall antioxidant capacity [7]; GSH-Px enzymatically neutralizes excess reactive oxygen species within cells, mitigating oxidative damage; and MDA levels indicate the rate and extent of lipid peroxidation, serving as an indirect marker of tissue oxidative injury; higher concentrations denote greater oxidative stress [24,25]. The absence of significant differences in T-AOC and GSH-Px across groups indicates that the antioxidant defense system was not consistently upregulated in response to the elevated MDA. This suggests that the oxidative stress observed in the MP group may reflect an imbalance between pro-oxidant and antioxidant forces at this intermediate nutritional level rather than a failure of the antioxidant system per se. Additionally, serum concentrations of growth-related hormones (GH, INS, IGF-1, and IGF-2) showed a declining trend with increasing concentrate nutritional levels across the LP, MP, and HP groups, consistent with the pattern observed for final body weight. Growth hormone is a master regulator of metabolism, stimulating hepatic gluconeogenesis, reducing lipogenesis while promoting lipolysis in adipose tissue, and enhancing protein synthesis. Insulin-like growth factors 1 and 2 act synergistically with GH to execute these anabolic functions [26]. Interestingly, some studies report that increasing dietary energy elevates the serum IGF-1 concentration in yaks [27], a finding that contrasts with our results. This discrepancy may be attributable to differences in the physiological state (e.g., gestation stage or production phase) of the experimental animals. Taken together, these results suggest that under warm-season housed conditions, strategic supplementation with low-nutritional-level concentrate feeds can effectively support the production performance of pregnant yaks.

### 4.3. Effects of Concentrate Supplementation on Rumen Microbial Community Composition in Pregnant Yaks

Ruminant nutrition research underscores the rumen’s paramount role as the primary digestive organ. Its resident microbial consortia ferment plant structural carbohydrates to generate volatile fatty acids (VFAs), vitamins, microbial protein, and other metabolites. VFAs alone are estimated to supply approximately 70% or more of the host’s daily metabolizable energy [28,29]. Therefore, fostering optimal rumen development and function is critical for maximizing nutrient utilization in ruminants. This study found that the rumen microbiota of yaks was predominantly composed of the phyla Bacillota (formerly Firmicutes) and Bacteroidota, a profile consistent with the typical rumen microbial ecology reported in other ruminant species [30,31,32]. Bacillota members are instrumental in degrading fibrous plant materials into absorbable short-chain fatty acids, whereas Bacteroidota are key players in carbohydrate metabolism and contribute to the development and regulation of the gastrointestinal immune system [33,34,35]. The numerically higher proportion of Firmicutes in the MP group compared with the LP and HP suggests that the medium-nutrition diet may have promoted the proliferation of fiber-degrading bacteria, potentially enhancing fiber digestion efficiency. Our analysis revealed no significant differences in Chao1 richness or Shannon diversity indices of the rumen microbial community with increasing concentrate nutritional levels. However, alpha diversity was numerically highest in the MP group. Furthermore, genera such as *norank_o_Clostridia_UCG-014*, *U29-B03*, and *Howardella* were significantly enriched in the rumen of the MP group compared with others, with *norank_o_Clostridia_UCG-014* exhibiting a notably higher relative abundance. Intriguingly, *norank_o_Clostridia_UCG-014* has been associated with blood glucose concentrations [36], and the *Rikenellaceae* family member *U29-B03* is implicated in promoting short-chain fatty acid production [37]. Spearman correlation and Mantel test analyses further delineated relationships between rumen microbial composition (at the genus level) and host blood metabolites. Blood GLU correlated positively with TC, HDL-C, and LDL-C; GH was positively correlated with INS, IGF-1, and IGF-2; and the overall genus-level microbial profile in the MP group correlated positively with host blood TG, TC, and HDL-C. These correlations support a functional linkage between the rumen microbial community and host lipid metabolism, particularly in the MP group. As noted, GH and IGFs are central regulators of anabolic metabolism [26]. Specifically, host blood TG and LDL-C concentrations were negatively correlated with the relative abundance of *Ruminococcus* and *Succiniclasticum* but positively correlated with *Olsenella*. *Succiniclasticum* is a specialist bacterium that converts succinate to propionate [38,39], a key gluconeogenic precursor in ruminants. Propionate, beyond its energetic role, may also enhance hepatic antioxidant enzyme expression via activation of peroxisome proliferator-activated receptors [40]. The enrichment of specific taxa in the MP group, coupled with its distinct correlation pattern with host lipid metabolites, suggests that the medium-nutrition diet induced a specific microbial shift that may have influenced host energy metabolism and redox status. However, the lack of corresponding changes in growth performance indicates that these microbial and metabolic modulations occurred within the homeostatic range of the animals. These interconnected findings suggest that concentrate supplementation at a medium nutritional level may remodel the host’s energy metabolism and redox balance networks by selectively enriching specific functional microbial taxa. This tailored microbial modulation appears to be a rational strategy for optimizing production performance in housed pregnant yaks. Although this study was limited by its sampling design, measured parameters, and sample size, which precluded a fully dynamic assessment across gestation, the results clearly indicate that appropriate concentrate supplementation can benefit the production performance of housed yaks in high-altitude regions.

Several limitations of the present study should be acknowledged. First, the sample size per group (*n* = 6) was relatively modest, which may have limited the statistical power to detect subtle differences in certain parameters, particularly for hormonal and antioxidant indicators that showed numerical but non-significant trends. Second, we did not measure fetal outcomes (e.g., calf birth weight, calf vitality, or immunoglobulin status) or postpartum maternal recovery, which are critical endpoints for evaluating the true efficacy of nutritional interventions during gestation. Long-term studies incorporating these parameters, as well as dynamic monitoring of rumen fermentation and metabolic flux across different gestational stages, are needed to comprehensively validate the optimal nutritional strategy for housed pregnant yaks. Finally, the 7-day adaptation period may be relatively short for complete rumen microbial adaptation to dietary changes. Although this duration was based on previous yak studies, longer adaptation periods have been recommended in recent research.

## 5. Conclusions

Based on the integrated assessment of performance, blood metabolites, and rumen microbiota, a low- to medium-nutrition concentrate appeared to be the most suitable range for housed pregnant yaks during the warm season. This level of supplementation effectively maintained growth performance, supported circulating concentrations of growth-related hormones, and regulated rumen microbial diversity without inducing excessive oxidative stress. However, the medium-nutrition (MP) group exhibited the highest serum MDA concentration (3.03 nmol/mL), which was significantly higher than that in the LP group, indicating that medium-nutrition supplementation was associated with increased lipid peroxidation. Therefore, while low- to medium-nutrition concentrates are sufficient to maintain production performance, the MP group should be approached with caution due to the potential for inducing oxidative stress. In summary, for housed pregnant yaks during the warm season, medium- to low-nutritional-level concentrate supplementation is sufficient to meet their gestational requirements and maintain production performance. These findings provide a scientific foundation for developing cost-effective and precise nutritional management strategies for pregnant yaks under housed conditions.

## Figures and Tables

**Table 1:** Composition and Nutritional Levels of Different Energy Supplement Feeds (Based on Dry Matter).

Items	Low-Nutrition Group	Medium-Nutrition Group	High-Nutrition Group
Ingredient Ratio (%)			
Corn	25.00	31.00	34.00
Corn germ meal	22.00	16.796	16.796
Wheat middlings	17.296	12.00	12.50
Corn DDGS ^1^	10.00	10.00	10.00
Soybean meal	9.00	8.00	9.00
Cottonseed meal	6.00	6.00	6.00
Rapeseed meal	3.00	6.00	5.50
Rice hull powder	3.00	5.50	1.50
Mountain flour	1.45	1.45	1.45
Calcium sulfate	1.10	1.10	1.10
Salt	1.00	1.00	1.00
Calcium phosphate dibasic	0.50	0.50	0.50
Complex trace elements ^2^	0.20	0.20	0.20
Montmorillonite	0.20	0.20	0.20
Mold inhibitor	0.075	0.075	0.075
Choline chloride	0.06	0.06	0.06
*Bacillus licheniformis*	0.025	0.025	0.025
Composite enzyme preparation	0.025	0.025	0.025
Comprehensive multivitamin ^3^	0.024	0.024	0.024
Betaine	0.02	0.02	0.02
Antioxidant	0.02	0.02	0.02
*Bacillus cereus*	0.005	0.005	0.005
Nutritional level (%) ^4^			
Digestive Energy (MJ/kg)	11.92	13.14	14.23
Crude protein	18.04	19.89	21.87
Crude fiber	17.46	11.62	7.09
Ether extract	4.49	4.31	4.41
Calcium	1.24	1.25	1.25
Phosphorus	0.60	0.61	0.64

^1^ Corn DDGS: Corn Distillers Dried Grains with Solubles. ^2^ Compound trace elements: Cu 27.68 mg/kg, Fe 259.83 mg/kg, Mn 79.32 mg/kg, Zn 149.28 mg/kg, I 1.09 mg/kg, Co 0.66 mg/kg, Se 0.27 mg/kg. ^3^ Compound Vitamins: VA 8.38 KIU/kg, VD_3_ 0.79 KIU/kg, VE 95.4 mg/kg, β-Carotene 3.15 mg/kg. ^4^ Digestive Energy was a calculated value, while the others were measured values.

**Table 2:** Effects of different nutritional levels of concentrate supplement on production performance and blood metabolites of pregnant yaks.

Items	LP	MP	HP	SEM	*p*-Value
Index of production performance					
IBW (kg)	228.17	228.42	226.75	11.050	0.998
FBW (kg)	290.92	288.17	288.75	6.741	0.986
ADG (kg/d)	0.57	0.54	0.56	0.051	0.977
DMI(kg/d)	5.58	5.42	5.15	0.186	0.669
F/G	11.51	11.96	10.87	1.216	0.942
Biochemical indices of blood					
GLU (mmol/L)	2.68	2.65	2.90	0.194	0.869
TG (mmol/L)	0.30	0.26	0.44	0.036	0.092
TC (mmol/L)	1.50	1.69	1.84	0.118	0.54
HDL (mmol/L)	1.05	1.26	1.28	0.104	0.638
LDL (mmol/L)	0.33	0.31	0.42	0.034	0.37
NEFA (mmol/L)	0.117	0.073	0.083	0.013	0.38
T-AOC (U/mL)	1.34	2.63	2.10	0.295	0.204
MDA (nmol/mL)	1.30 ^b^	3.03 ^a^	1.94 ^ab^	0.290	0.036
GSH-Px (U/mL)	75.97	63.14	49.06	6.185	0.214
GH (ng/mL)	2.97	2.40	1.98	0.198	0.119
INS (mIU/L)	4.64	4.39	3.52	0.333	0.375
IGF-1 (ng/mL)	48.90	44.19	32.67	4.083	0.261
IGF-2 (ng/mL)	7.06	6.63	5.06	0.447	0.159

Values are presented as means, (*n* = 6). SEM, standard error of the mean. BW, body weight; ADG, average daily gain; DMI, average daily feed intake; F/G, feed-to-gain ratio; GLU, glucose; TG, triglycerides; TC, total cholesterol; HDL-C, high-density lipoprotein cholesterol; LDL-C, low-density lipoprotein cholesterol; NEFA, non-esterified fatty acids; T-AOC, total antioxidant capacity; MDA, malondialdehyde; GSH-Px, glutathione peroxidase; GH, growth hormone; INS, insulin; IGF, insulin-like growth factor. ^a,b^ Means within a row with different superscript letters differ significantly (*p* < 0.05).

## Data Availability

Raw sequencing data have been deposited at the NCBI Sequence Read Archive (SRA) under BioProject ID PRJNA761685.

## Supplementary Materials

The following supporting information can be downloaded at https://www.mdpi.com/article/10.3390/microorganisms14071506/s1.

## Abbreviations

The following abbreviations are used in this manuscript:

MDA	Malondialdehyde
GH	Growth hormone
INS	Insulin
IGF-1	Insulin-like growth factor 1
IGF-2	Insulin-like growth factor 2
TG	Triglycerides
TC	Total cholesterol
HDL-C	High-density lipoprotein cholesterol
LDL-C	Low-density lipoprotein cholesterol
LEfSe	Linear discriminant analysis effect size
FBW	Final body weight
IBW	Initial body weight
ADG	Average daily gain
DMI	Dry matter intake
F/G	Feed-to-gain ratio
GLU	Glucose
NEFA	Non-esterified fatty acids
T-AOC	Total antioxidant capacity
GSH-Px	Glutathione peroxidase
VFA	Volatile fatty acid
DE	Digestible energy
